# Construction of Brazil nut (*Bertholletia excelsa: Lecythidaceae*) cambium normalized cDNA libraries for 454 next generation sequencing

**DOI:** 10.1186/1753-6561-5-S7-P4

**Published:** 2011-09-13

**Authors:** Peter Inglis, Ana Ciampi, Vânia Azevedo

**Affiliations:** 1 Laboratório de Genética Vegetal, Embrapa Genetic Resources and Biotechnology, Brazilia, Brazil

## Background

The Brazil nut, *Bertholletia excelsa* (Lecythidaceae), is the most important non-timber forest resource in the Amazonian biome, where nuts for the international trade are exclusively wild-collected. The monotypic genus possesses a discontinuous distribution and is thought to have rapidly and recently radiated south and westward from a northern/eastern Amazonian origin, largely as a result of ancient human activity [1]. It's genetic variability is currently thought to be low and conserved in one or very few populations [2], and in order to better understand the population genetics of this tree, there is a need for additional, or more informative, molecular markers than is currently available.

## Methods

Trunk cambium is an attractive source of material for cDNA library construction, in contrast to foliar material, where photosynthetic genes are grossly overrepresented. Furthermore, samples can be taken from mature trees at the forest floor level. Cambium samples were collected from both Acre and Amapá states of Brazil, and from both highly, and poorly-productive trees from the respective states, with the aim of detecting microsatellites (SSRs) and single nucleotide polymorphisms (SNPs) for genetic analysis. The samples were frozen in the field on dry ice and later kept at -80 °C in the laboratory. Total RNA was extracted from cambium ground with a pestle and mortar under liquid nitrogen, using either the RNeasy kit (QIAGEN) or a combination of hot CTAB pre-treatment with repeated chloroform extraction and the RNeasy kit. Messenger RNA was purified using oligo dT-magnetic bead separation (Dynabeads; Invitrogen). The Clontech SMART kit and Superscript III (Invitrogen) reverse transcriptase were used for cDNA synthesis and four normalized, PCR-amplified libraries constructed using the Trimmer Direct kit (Evrogen). The four cDNA libraries were multiplex tagged and submitted to a single 454 pyrosequencing production run, using a GS FLX-Titanium instrument.

## Results and conclusions

Cambium RNA extracted with the RNeasy kit alone or with the CTAB pretreatment possessed A260/280 and A260/230 ratios above 2.0, when measured by Nanodrop spectrometry, indicating high purity, and appeared to be of high quality by agarose electrophoresis. However, the RNA extracted by RNeasy alone was not successfully reverse-transcribed and gave no significant products in RT-PCR, even after mRNA purification by magnetic bead separation, indicating the presence of recalcitrant and bound enzyme inhibitory substances. In contrast, the CTAB pretreatment yielded RNA that could be readily reverse transcribed, which was subsequently used for amplified and normalized cDNA library construction.

Data yield for the four normalized libraries averaged 73 million total bases, 270 thousand validated sequences with a mean read length of 265 bp, respectively, per library. Sequences were assembled into contigs using Newbler version 2.5 and microsatellites were searched for using Microsatellite Commander v. 0.8.2. The mean numbers of microsatellites identified per repeat class per library were: 507 dinucleotide, 897 trinucleotide, 29 tetranucleotide, 9 pentanucleotide, 41 hexanucleotide, and 138 compound/interrupted repeats, with little variation in microsatellite yield or repeat class present between libraries. These libraries appear to be a rich source of new markers, which will greatly enhance our efforts to better understand the population genetics of *B. excelsa*, to determine appropriate conservation strategies, and to work towards rational selection of productive lines.
